# Healthy Living after Cancer: a dissemination and implementation study evaluating a telephone-delivered healthy lifestyle program for cancer survivors

**DOI:** 10.1186/s12885-015-2003-5

**Published:** 2015-12-21

**Authors:** Elizabeth G. Eakin, Sandra C. Hayes, Marion R. Haas, Marina M. Reeves, Janette L. Vardy, Frances Boyle, Janet E. Hiller, Gita D. Mishra, Ana D. Goode, Michael Jefford, Bogda Koczwara, Christobel M. Saunders, Wendy Demark-Wahnefried, Kerry S. Courneya, Kathryn H. Schmitz, Afaf Girgis, Kate White, Kathy Chapman, Anna G. Boltong, Katherine Lane, Sandy McKiernan, Lesley Millar, Lorna O’Brien, Greg Sharplin, Polly Baldwin, Erin L. Robson

**Affiliations:** The University of Queensland, School of Public Health, Brisbane, Australia; Queensland University of Technology, School of Public Health and Social Work, Institute of Health and Biomedical Innovation, Brisbane, Australia; University of Technology Sydney, Centre for Health Economics Research and Evaluation, Sydney, Australia; The University of Sydney, Concord Clinical School, Sydney, Australia; Mater Hospital Sydney, The Patricia Ritchie Centre for Cancer Care and Research, Sydney, Australia; Swinburne University of Technology, School of Health Sciences, Melbourne, Australia; Peter MacCallum Cancer Centre, Department of Cancer Experiences Research, Melbourne, Australia; Sir Peter MacCallum Department of Oncology, University of Melbourne, Melbourne, Australia; Flinders Medical Centre, Department of Medical Oncology, Bedford Park, Australia; University of Western Australia, School of Surgery, Perth, Australia; University of Alabama at Birmingham Comprehensive Cancer Center, Birmingham, USA; University of Alberta, Faculty of Physical Education and Recreation, Edmonton, Canada; University of Pennsylvania, Perelman School of Medicine, Center for Clinical Epidemiology and Biostatistics, Philadelphia, USA; Centre for Oncology Education and Research Translation (CONCERT), Ingham Institute for Applied Medical Research, South Western Sydney Clinical School, UNSW Medicine, University of New South Wales, Sydney, Australia; University of Sydney, Sydney Nursing School, Sydney, Australia; Cancer Council New South Wales, Sydney, Australia; Cancer Council Victoria, Melbourne, Australia; Melbourne School of Health Sciences, The University of Melbourne, Melbourne, Australia; Cancer Council Western Australia, Perth, Australia; Cancer Council South Australia, Adelaide, Australia

**Keywords:** Lifestyle intervention, Cancer survivors, Dissemination and implementation study, Physical activity, Nutrition

## Abstract

**Background:**

Given evidence shows physical activity, a healthful diet and weight management can improve cancer outcomes and reduce chronic disease risk, the major cancer organisations and health authorities have endorsed related guidelines for cancer survivors. Despite these, and a growing evidence base on effective lifestyle interventions, there is limited uptake into survivorship care.

**Methods/Design:**

Healthy Living after Cancer (HLaC) is a national dissemination and implementation study that will evaluate the integration of an evidence-based lifestyle intervention for cancer survivors into an existing telephone cancer information and support service delivered by Australian state-based Cancer Councils. Eligible participants (adults having completed cancer treatment with curative intent) will receive 12 health coaching calls over 6 months from Cancer Council nurses/allied health professionals targeting national guidelines for physical activity, healthy eating and weight control. Using the RE-AIM evaluation framework, primary outcomes are service-level indicators of program reach, adoption, implementation/costs and maintenance, with secondary (effectiveness) outcomes of patient-reported anthropometric, behavioural and psychosocial variables collected at pre- and post-program completion. The total participant accrual target across four participating Cancer Councils is 900 over 3 years.

**Discussion:**

The national scope of the project and broad inclusion of cancer survivors, alongside evaluation of service-level indicators, associated costs and patient-reported outcomes, will provide the necessary practice-based evidence needed to inform future allocation of resources to support healthy living among cancer survivors.

**Trial registration:**

Australian and New Zealand Clinical Trials Registry (ANZCTR) - ACTRN12615000882527 (registered on 24/08/2015)

## Background

### Cancer survivorship in Australia

Five-year survival rates for the majority of cancers have steadily improved in Australia over the past decades, from 47 % to 66 % between the periods 1982–1987 and 2006–2010, with rates for the most prevalent cancers - prostate, breast and colorectal cancers - now being 92 %, 89 % and 66 %, respectively [1, 2]. These improvements in survival are also mirrored across other developed countries [3]. However, cancer survivorship brings with it increased risk of cancer recurrence, second primaries, persistent side-effects of treatment, functional decline and co-morbid chronic conditions, such as cardiovascular disease and type 2 diabetes [4–7]. To mitigate these risks and improve the longer-term well-being of cancer survivors, national cancer organisations recommend that cancer survivors engage in regular physical activity, eat a healthy diet and keep their weight within a healthy range [8–10]. Yet, the majority of cancer survivors do not meet these lifestyle recommendations; over 50 % are overweight or obese, over 50 % do not meet physical activity recommendations, and 90 % do not meet dietary guidelines [11–13]. Further, declines in activity and weight gain are common post-cancer treatment [14–16]. Despite a strong desire on the part of most cancer survivors for advice and support regarding healthy lifestyles [17], such assistance is not routinely offered as part of survivorship care [17–19].

### Lifestyle interventions for cancer survivors

Initial research in lifestyle interventions in cancer survivors emphasised efficacy trials of highly selected participants (primarily women with early stage breast cancer), with strictly controlled intervention protocols delivered in supervised, clinic settings [20–25]. Over the past decade, the number of trials of lifestyle interventions in cancer survivor groups other than breast has expanded, demonstrating their safety, feasibility and efficacy in gynaecological cancers, prostate, colorectal, and lung cancers [26–42]. With the efficacy of such interventions, particularly for physical activity, firmly established [43–45], the emphasis has shifted to effectiveness or pragmatic trials designed to answer questions important to informing translation into routine practice (How do we reach the growing number of cancer survivors, many of whom live outside of metropolitan areas? Which health care providers are best suited to deliver lifestyle interventions, and what are the resources required for intervention delivery? Are post-intervention improvements in patient-reported outcomes maintained or are booster sessions needed?) [46–49].

A particular area of interest has been the evaluation of broad-reach or distance intervention modalities, particularly the telephone, as this mode of delivery has the potential for far greater reach compared to face-to-face delivered interventions [32, 34, 35]. A recent systematic review of 27 trials of broad-reach lifestyle interventions among cancer survivors (22 of them telephone-delivered) found evidence for improvements in lifestyle behaviours and weight loss across cancer survivor groups [50]. In a comparative-effectiveness trial of an exercise intervention for women during and after treatment for breast cancer, a telephone-delivered intervention was as effective as a face-to-face-delivered intervention for achieving improvements in fitness and quality of life [51, 52] and suitable for reaching women living in regional and rural Australia [53]. This now large body of evidence on the efficacy and effectiveness of lifestyle interventions among diverse cancer survivors, including evidence for the effectiveness of telephone delivery, sets the stage for the present study and a future mechanism of routine support for cancer survivors.

### The Healthy Living after Cancer partnership

Healthy Living after Cancer (HLaC) is a dissemination and implementation study that will evaluate the integration of an evidence-based lifestyle intervention for cancer survivors into an existing telephone cancer information and support service delivered by the Australian state-based Cancer Councils. Outcomes, as described in detail below, are service-level and consumer/patient-reported.

In Australia, the Cancer Councils are non-governmental, not-for-profit, cancer control organisations located in each state and territory that deliver programs and provide information and support across the cancer continuum from cancer prevention through to cancer survivorship. They also fund as well as conduct research. The Cancer Councils offer a telephone “13 11 20 Cancer Information and Support Service” (previously known as the Helpline) with coverage across Australia. This existing service delivery infrastructure, combined with the priority placed on survivorship support, and their position stand on nutrition and physical activity for cancer survivors [9], provide an optimal framework on which to scale-up, implement nationally and evaluate an evidence-based lifestyle intervention for cancer survivors.

The five largest Cancer Councils were approached for collaboration, with four agreeing to take part (Cancer Councils New South Wales, Victoria, South Australia and Western Australia). The Australian National Health and Medical Research Council Partnership Projects scheme, which is designed to support academic-industry research partnerships leading to translation of results into health policy and practice [54], was targeted for funding. A concept development workshop was organised to engage cancer policy and practice stakeholders, including the Cancer Councils, along with national and international lifestyle and cancer survivorship experts and clinicians (co-authors on this paper). The workshop was sponsored by two Australian Cancer Trials Groups, which led to the development of a funding proposal, and the subsequent collaboration that now governs the Healthy Living after Cancer Partnership Project.

The five-year study will be conducted in three phases: Phase 1 involves knowledge transfer and capacity building (Year 1). In this phase, the research team will work closely with Cancer Council partners to facilitate their ability to implement and evaluate HLaC, including database adaptations, staff training and input from Cancer Council staff on program materials. Phase 2 involves HLaC implementation and evaluation (Years 2–5), with intervention procedures and the evaluation plan described below. Phase 3 involves advocacy within the Cancer Councils, alongside communication of HLaC outcomes to the broader cancer control community and health care sector, in an effort to obtain sustained funding for the program (Years 4–5).

## Methods/Design

### Study design

The HLaC dissemination and implementation study uses a single-group, pre-post-test study design. The research questions to be answered are those important to informing translation into practice, particularly the feasibility and costs associated with wide-scale program implementation [46, 55] and the cancer survivor outcomes that can be achieved in the health services delivery context. Accordingly, the RE-AIM Framework (reach, effectiveness, adoption, implementation, maintenance) [56] is used to guide systematic evaluation, with assessment of: the *reach* of the intervention (i.e., the number and representativeness of service referrers and participants/consumers); program *implementation* (i.e., number of referrals, consent rates, fidelity of delivery, program completion rates and costs to deliver); *effectiveness* (i.e., cancer survivor outcomes including behavioural, anthropometric and psychosocial variables); and *maintenance* (i.e., the capacity of the Cancer Councils to continue to deliver the service following the end of this study). Ethical approval was granted from the human research ethics committees of the following institutions: Cancer Council Victoria (on behalf of Cancer Councils Victoria and South Australia), Cancer Council New South Wales, the University of Queensland and the University of Western Australia (on behalf of Cancer Council Western Australia). Ethical approval is also sought as required for referring clinical sites in these states.

### Participants and referral pathways

The HLaC program is targeted to any adult cancer survivor who has been treated with curative intent and who has completed treatment. Referral pathways for potentially eligible participants fall under three broad headings: Cancer Council services and programs (e.g., the 13 11 20 information and support service; survivorship support and education programs); Cancer Council networks and partners (e.g., Prostate Cancer Foundation of Australia; Breast Cancer Network Australia); and cancer treatment sites (e.g., metropolitan and regional hospitals). Within these categories, each Cancer Council will develop, in conjunction with the research team, its own HLaC referral pathways based on local knowledge and with a study-wide agreed emphasis on outreach to non-urban areas in which 30 % of Australian cancer survivors reside [57].

Cancer Council staff will provide representatives at each referring organisation or site with information about the HLaC program (a referrer information sheet), along with promotional material (posters and brochures), and will be encouraged to recommend the program to any potentially interested and eligible patients and cancer survivors. Based on varying ethics requirements across sites, at some, interested patients will give verbal consent for their name and contact details to be forwarded to their local Cancer Council so that HLaC project staff may telephone them to discuss the program in more depth (including screening for eligibility). At other sites, interested patients will be given a referral form to return to their Cancer Council. Interested cancer survivors may also self-refer by telephoning 13 11 20 and enquiring directly about the program at any time, as advertised on the promotional posters and brochures.

### Accrual targets

The overarching aim is for each Cancer Council to deliver the program over a three-year period. This should afford adequate experience with program implementation and familiarity with outcomes to inform decisions on program sustainability. Accrual targets for each Cancer Council are based on available resources to implement the program. There is capacity for each participating Cancer Council to achieve approximately 50–60 program completions/year implementing the HLaC program 6-month/12 call protocol (refer to the program delivery section). With an estimated 30 % attrition, each Cancer Council would therefore need to enrol approximately 75–80 participants per year. The attrition estimate is based on the high (nearly 50 %) attrition observed in other disseminated lifestyle intervention programs in the general adult population [58, 59], but tempered by the fact that cancer survivors tend to be highly motivated such that cancer-specific trials generally achieve 90 % 12-month retention [32, 35, 52, 53, 60]. Across the three years of HLaC implementation, it is anticipated that each Cancer Council will achieve completion rates of approximately 150–180 participants, for a total of approximately 600–680 HLaC completing (from 900 enrolled) during the proposed study (refer to the sample size section).

### Screening and consent

The eligibility criteria for the HLaC program are designed to be as broad as possible, thereby maximising the diversity of survivors who are able to take part and ensuring participants are able to do so safely. The eligibility criteria are: adults (18+ years); diagnosed with localised (i.e., non-metastatic) cancer of any type treated with curative intent; completed primary treatment (i.e., surgery, chemotherapy, radiation; patients currently receiving hormonal treatment or Herceptin are still eligible); no contraindications to engaging in unsupervised physical activity (i.e., active heart disease, breathing problems requiring hospitalisation in the past 6 months, undergoing dialysis, diabetic complications such as severe neuropathy or retinopathy, planning a knee or hip replacement in the next six months, pregnant); no cognitive or mental health impairments that would hinder program participation; able to speak and read English sufficiently to allow for program participation; wanting support for healthy living via physical activity and healthy eating and willing to make a six-month commitment to HLaC program participation. Screening will occur over the telephone by Cancer Council staff utilising a recruitment and screening script, with eligibility based on patient self-report (Table 1). In cases where eligibility is uncertain, the patient will be asked to seek permission to take part from their treating clinician and report back to Cancer Council staff, with the outcome recorded in the database. Those eligible will be posted an information sheet and a follow-up telephone call will be completed a week later to obtain informed consent (verbal and audiotaped or paper-based, depending upon the recruitment protocol used at the referring site).

**Table 1:** Eligibility criteria and their associated screening questions

Eligibility criteria	Screening question/s
Adults aged 18+ years	• What is your date of birth (day, month and year)?
Diagnosed with localised potentially curative cancer of any type	• When were you diagnosed with cancer (most recent diagnosis)? Please tell me the day, month and year as best you can remember. • What type of cancer were you diagnosed with? • Was your cancer localised, or did it spread to other parts of your body (i.e., were you diagnosed with metastatic disease or advanced cancer)?
Completed treatment (i.e., surgery, chemotherapy, radiation; hormonal treatment or Herceptin are fine)	• Have you completed treatment for cancer – i.e., surgery, chemotherapy or radiation therapy? This does not include hormonal treatment or Herceptin which you may still be on.
Without contraindications to engaging in unsupervised physical activity	• Are you currently pregnant or lactating or intending to become pregnant in the next 6 months? • Do you currently use a walker or wheelchair regularly to help you walk or move around? This does not include using a walking stick. • Have you had any health problems, such as a stroke, or have you had an accident, that has left you with walking difficulties? • Do you have plans for a hip or knee replacement in the next 6 months? • Do you ever feel any pain, tightness or heaviness in your chest either when you are resting or when you are physically active? • Have you been told by your doctor that you have a heart condition and that you should only do physical activity supervised by a health professional? • Have you been told by your doctor that you’ve had a heart attack within the last 6 months? • Have you had any breathing problems that required hospitalisation or oxygen use within the past 6 months? • Do you have severe chronic lung disease? • Do you take the blood thinners Warfarin, Coumadin or Marevan? • Do you have moderate to severe kidney disease or are you undergoing dialysis? • Do you suffer from neuropathy or nerve damage, which is most commonly caused by complications from diabetes? • Do you suffer from retinopathy or damage to the retina in the eye, most commonly caused by complications from diabetes?
Without cognitive or mental health impairments that would hinder program participation	• Have you ever been diagnosed with depression/anxiety/any other mental health condition? • If yes, are you currently suffering from depression/anxiety/any other mental health condition? • If yes, is your depression/anxiety/any other mental health condition currently stable and/or being managed by medication or treatment from a health professional?
Able to speak and read English sufficiently to allow for program participation	Assessed by staff during the screening call (i.e., is the person they are screening able to understand the questions and respond appropriately).
Wanting support for healthy living via exercise and healthy eating and willing to make a six-month commitment to HLaC program participation	Participants are asked to read the Participant Information Sheet and consider whether now is a good time for them to take part in the program before providing consent.

### Healthy Living after Cancer program

The six-month HLaC program is aimed at increasing physical activity, promoting healthy eating, and assisting with moderate weight loss (if indicated), consistent with current evidence and guidelines for nutrition and physical activity in cancer survivors (Table 2) [8–10]. Delivered by study-trained, Cancer Council nurses/allied health professionals with expertise in cancer care, it will provide the necessary support and advice to meet these guidelines while dealing with common cancer survivorship issues including lymphoedema, pain, fatigue and fear of recurrence. Participants will receive up to 12 telephone calls over the 6-month program along with a HLaC Participant Workbook (Table 3) used to support intervention delivery.

**Table 2:** Healthy Living after Cancer intervention targets

Maintain a healthy body weight (BMI between 18.5 – 24.9 kg/m^2^)	
Engage in 30 minutes or more of moderate physical activity daily	
Consume a low saturated fat diet, including 5 serves of vegetables and 2 serves of fruit daily

**Table 3:** Healthy Living after Cancer Participant Workbook content

Introduction to Healthy Living after Cancer (Section 1) • Importance of healthy living for cancer survivorship	
Plan for Success (Section 2) • Participant aims • SMART goal setting • Problem solving • Tracking your progress	
Physical Activity (Section 3) • Aerobic activity (e.g., brisk walking) • Flexibility/stretching • Strength training • Reducing sitting time	
Healthy Eating (Section 4) • Increasing serves of vegetables, fruit and wholegrains • Reducing fat (particularly saturated fat) intake • Eating the right amount of food (portion control) • Choosing the right types of food (low calorie, high nutrient)	
Weight Loss/Maintenance (Section 5) • Setting an appropriate weight loss goal (5 % – 10 % of initial body weight) • Strategies for achieving a 2000 kJ/day reduction	
Staying on Track (Section 6) • Positive thinking • Getting back on track after a slip • Planning ahead • Getting support from others • Celebrating success	

Cancer Council nurses/allied health professionals will be trained in the HLaC protocol by study investigators. A Training Manual detailing the intervention protocol, including example call scripts, will be made available to all trainees prior to a two-day, group-based training workshop, which makes extensive use of role-playing. Using a train-the-trainer approach, the training is video-taped and a lead nurse from each Cancer Council will take responsibility for the initial training of any new staff requiring training during the study period. Within each Cancer Council, those delivering the program will debrief weekly on participant progress with the lead HLaC nurse, alongside email access to the research team for support as needed and monthly case management teleconferences organised with the lead investigator.

Intervention delivery is grounded in Social Cognitive Theory constructs of self-efficacy, social support and outcome expectancies [61] and is guided by techniques of motivational interviewing [62] and health behaviour coaching [63]. The emphasis is on developing participant skills in evidence-based behaviour change strategies – goal setting, self-monitoring, problem solving, identifying social support, stimulus control, positive self-talk and self-reward [64]. The structured protocol for each call includes: assessment of progress; problem-solving; advice/education; and collaborative (“SMART”) goal-setting/goal progression (detailing a behaviourally-specific plan for goal achievement).

#### Intervention procedures

A semi-structured approach to the order in which intervention targets are addressed is used to guide delivery and works in accordance with the Participant Workbook (see Table 3). Participants are encouraged to begin with a focus on increasing physical activity, as it is often more challenging to achieve than making small changes to dietary intake. However, consistent with the motivational interviewing approach, the intervention is tailored to each participant, with an initial focus on targets in areas that the participant is most motivated and confident to change. The intervention is delivered in three phases, with weekly, fortnightly and then monthly calls, as depicted in Table 4.

**Table 4:** Intervention phases, call frequency and call objectives

Phase	Call frequency	# Calls	Purpose	Objectives
Phase one Month 1	Weekly	1-4	Rapport-building, engagement, education, skill-building	• Program overview • Feedback on pre-program assessment to build motivation to change • Build engagement through homework and self-monitoring • Understand importance of physical activity, healthy eating and healthy weight • Understand and begin using behaviour change skills: setting goals, tracking, problem-solving, identifying benefits, rewarding success
Phase two Months 2-3	Fortnightly	5-8	Putting it into practice	• Progress goals • Add new target behaviours • Review progress, reinforce success, identify benefits and problem-solve barriers • Ongoing education
Phase three Months 4-6	Monthly	9-12	Consolidation and maintenance	• Use of supports and strategies for maintaining changes • Shift to participant lead in progressing goals and tracking behaviour • Closure

#### Physical activity

The physical activity component of the intervention focuses on identifying enjoyable activities that can be easily incorporated into a participant’s lifestyle (e.g., walking), with gradual increases in physical activity aimed at meeting or exceeding the target of 30 minutes per day of moderate-to-vigorous intensity activity daily. Resistance exercise (2–3 sessions/week) is also encouraged, with detailed photographs and instructions, guidelines on the number of sets and repetitions of each exercise, and options for progression, outlined in the Participant Workbook. In addition to daily planned physical activity, participants are encouraged to capitalise on opportunities to be active in and around their homes and workplaces (e.g., gardening, housework, taking the stairs) [65–67] and to reduce sitting time (i.e., to get up and move every 30 minutes and to aim for no more than 2 hours/day of screen time outside of work) [68–70].

#### Diet and weight loss

All participants are encouraged to achieve three overarching dietary aims: 1) increasing intake of vegetables, fruit and whole grains; 2) reducing intake of foods high in added sugars and fat (especially saturated fats) and 3) limiting portion size and improving dietary quality. In addition, those who are overweight and want to work towards modest weight loss (i.e., 5–10 % of initial body weight) focus on reducing energy intake by 2,000 kJ per day. Strategies to reduce energy intake include: improving portion control (by reducing portion size or number of serves) and lowering energy density (by increasing intake of low energy dense foods such as fruits and vegetables and reducing intake of foods with high energy density such as high fat/sugar foods).

### Data collection

All study data are collected by study-trained Cancer Council staff as this enables capacity building for ongoing program evaluation. Study funding is used to ensure that each Cancer Council has a dedicated research assistant to support evaluation. Study protocol implementation and data quality control are monitored via weekly database reports submitted by Cancer Council staff to the research team.

### Primary and secondary outcomes

Outcomes are shown in Table 5, along with the relevant RE-AIM indicators and measurement tools. Primary (service-level) outcomes include referrals, call delivery, completion rates, participant and staff satisfaction and program delivery costs, and will be systematically collected in each of the Cancer Council databases. Secondary (anthropometric, behavioural and psychosocial) outcomes are patient-reported during pre- and post-program assessments conducted by Cancer Council staff via telephone and using validated protocols and questionnaires.

**Table 5:** Primary and secondary outcomes, assessment tools and RE-AIM indicators

Setting	RE-AIM indicator	Collection method/assessment tools
Primary Outcomes		
Referring Sites	Adoption & Maintenance	
	Type of referring site and # of referrals	CC database
	Staff satisfaction and feedback on sustainability of referral protocol	Interview
Cancer Councils	Reach & Representativeness	
	% uptake among eligible survivors	CC database
	Participant characteristics	CC database
	Implementation	
	Participant consent rates	CC database
	Completion rates	CC database
	Withdrawal rates and reasons	CC database
	Number of intervention calls	CC database
	Length of intervention calls	CC database
	Administration time for intervention calls	CC database
	Intervention content covered	CC database
	Completion of pre- and post-program assessments	CC database
	Adverse events	CC database
	Healthy Living after Cancer nurse/allied health professional and CC manager satisfaction	Interview
	Economic Appraisal	
	Costs to deliver the Healthy Living after Cancer program	Documentation of resources utilised for Healthy Living after Cancer service delivery
Secondary Outcomes		
Consumer	Effectiveness	
	Anthropometric Outcomes	
	Weight, height, waist circumference	Self-reported
	Behavioural Outcomes	
	Physical Activity	Active Australia Survey [83]
	Sedentary Behaviour	Single item from the International Physical Activity Questionnaire (short, last 7 days format) [84]
	Diet	Fat and Fibre Behaviour Questionnaire [85]
		Daily servings of fruits and vegetables [86]
	Psychosocial Outcomes	
	Quality of Life	Short-Form Health Survey (SF-12), v1.0 [87]
	Cancer and treatment-related symptoms and side-effects	MD Anderson Symptom Inventory [88]
	Fear of Cancer Recurrence	The Concerns about Recurrence Questionnaire – 4-item (CARQ-4) [89]
	Distress	2-item Distress Thermometer assessing distress (adapted from the National Comprehensive Cancer Network Distress Thermometer for Patients) [90] and impact of distress [91]
	Healthy Living after Cancer program satisfaction	Self-reported (rating scales and comments)

*CC* Cancer Council

### Statistical analyses

Primary outcomes for HLaC implementation will be reported descriptively. Analyses of secondary (effectiveness) outcomes will be by mixed models, which allow for repeated measures (baseline and follow-up) and will include all participants with baseline data (including those with missing data at follow-up) with adjustment for predictors of dropouts to minimise non-response bias. Data will be analysed collectively (pooled) across Cancer Councils as well as reported individually for each. To minimise type I errors because of testing multiple outcomes, significance will be set at *p* < 0.001 (two-tailed). Sensitivity of conclusions to missing data assumptions will be evaluated.

### Sample size related to secondary (effectiveness) outcomes

Given the resources available in each Cancer Council to support HLaC implementation, the service delivery is expected to provide a sample size of approximately 900 participants across the four participating Cancer Councils over the three year recruitment period. This sample provides >90 % power with two-tailed significance of *p* < 0.001 to detect pre-post changes of 60 minutes of physical activity per week, 0.5 serves of fruit or vegetables, 2 kg weight and clinically relevant changes in physical and mental components of quality of life (3 units each) [71], based on assumed standard deviations of change of 300 min/week, 1.5 fruit and 2 vegetable serves, 8 kg weight, and 8 units on the mental and physical component scores. These calculations allow for 30 % participant attrition and adjustment for up to 10 covariates (10 observations per covariate).

### Economic appraisal

The economic questions associated with this research relate to the costs of implementing HLaC and the relationship between costs, program completion and outcomes. Specifically:

- *Costs of implementing HLaC:* The fixed costs (i.e., expenditure required to deliver the HLaC program, including resources associated with modifying Cancer Council databases, refining referral pathways, adapting the intervention and evaluation protocols and recruiting and training nurses) will be documented. Dollar values will be attached to these resources using publicly available information such as appropriate salary rates for the time of personnel involved in the above activities and commercial prices for the production of any training materials, etc. Fixed costs will be allocated equally over all participants who consent to participate in HLaC. Variable costs (i.e., those that are proportional to the volume of service provided) will be allocated in proportion to the stage of HLaC reached by individuals [72].
- *Relationship between costs, completion and outcomes:* Stage of completion will be defined as follows: Stage 1 = completion of ≥3 x weekly phone calls; Stage 2 = completion of Stage 1 + ≥3 x fortnightly phone calls; Stage 3 = completion of stages 1 + 2 + ≥3 monthly calls. For the purposes of the economic appraisal, outcomes will be defined using a series of pre-defined benchmarks based on pre-post program changes in the behavioural and anthropometric variables. For total physical activity, participants who reported achieving 150 minutes/week are considered as having made improvements in their physical activity level. For vegetables and fruit, participants who reported achieving the target of 5 and 2 serves/day, respectively, are classified as having made positive changes in their dietary behaviour. For weight (among participants where weight loss is indicated), those who recorded a body weight reduction at follow-up ≥3 % of their baseline weight will be classified as having successfully achieved a health enhancing benefit [73]. Regression analysis will be used to assess the relationship between completion of stages and outcomes achieved. It is hypothesised that those who complete Stage 3 will have better outcomes than Stage 2 completers who in turn will have better outcomes than those who do not proceed beyond Stage 1. Pre- and post-test differences will be calculated as changes in Quality of Life (SF-12) and Quality Adjusted Life Years (QALYs) SF6D [74] (calculated from SF-12). Data will be analysed with and without imputed outcomes. Sensitivity analysis will be used to explore the impact of varying assumptions about the cut-offs used to define improvements in the secondary outcomes noted above.

## Discussion

This study represents the first scaled-up and national-level implementation and evaluation of an evidence-based lifestyle intervention for cancer survivors in collaboration with a peak cancer control and community facing partner. As such, it is consistent with calls for the conduct of practice-based and dissemination research that accelerates the transfer of cancer survivorship research into evidence-based cancer care [47–49]. A participatory and collaborative approach has been used to build both capacity amongst Cancer Councils for program delivery and evaluation and to ensure the collection of outcomes data necessary to inform decisions about sustained funding. Accordingly, from the outset, the study has engaged a group of cancer policy, practice and service delivery stakeholders, along with lifestyle and cancer survivorship experts and cancer clinicians. This transdisciplinary collaboration provided guidance on study design, evaluation and intervention protocol adaptation and will oversee study implementation, culminating in consideration of and advocacy around study findings in relation to sustained funding for HLaC program delivery.

As a dissemination and implementation study there are some inherent limitations. The use of a single group, pre-post study design is primary among these. However, the primary questions to be answered are about the feasibility and costs of wide-scale implementation, and the survivor outcomes that can be achieved in this context. Numerous previous efficacy trials have answered the question as to whether lifestyle intervention is superior to usual care or no intervention [43–45, 75–77]. The use of self-report measures of health behaviour changes is a limitation [78, 79], however, all self-reported tools have been validated, including against objective measurement where feasible [80–82], and have been used in cancer samples, with the national scale of implementation and resource limitations precluding collection of clinically-assessed outcomes. The collection of data by Cancer Council staff, some of whom will have a role in program delivery, may be a source of bias. However, in this context, it was felt that (i) the emphasis on building evaluation capacity among the Cancer Council partners outweighed any inherent bias and (ii) this protocol is more closely representative of the real-world process of program delivery and evaluation, with both important for supporting the final phase of the project, specifically, advocating for sustained HLaC delivery beyond the funding period.

It is important to note that the healthy living targets being promoted in this study, and ratified by Cancer Council Australia and the World Cancer Research Fund, are based on general population recommendations for cancer prevention [8–10]. And further, that the vast majority of lifestyle intervention evidence among cancer survivors has been conducted in healthier samples with early (not advanced) cancers. Accordingly, the HLaC study targets survivors treated with curative intent. There is still more research needed to inform the safety, feasibility and efficacy of lifestyle interventions for those with advanced cancers, as well as understanding the upper and lower thresholds for physical activity, dietary change, and weight loss, and the optimal sequencing of these multiple lifestyle intervention targets.

The HLaC study is national in scope, involving the relevant cancer control stakeholders and multidisciplinary expertise; is based on broad inclusion of cancer survivors; and includes an evaluation of both service-level indicators and associated costs and cancer survivor outcomes. The evidence to be generated from this collaborative study is what is directly required to influence health policy and practice related to cancer survivorship care around provision of support for healthy living after cancer.
